# Three Dimensional Culture of Potential Epithelial Progenitor Cells in Human Lacrimal Gland

**DOI:** 10.1167/tvst.8.4.32

**Published:** 2019-08-30

**Authors:** Hui Lin, Ying Liu, Samuel Yiu

**Affiliations:** 1 Wilmer Eye Institute, School of Medicine, Johns Hopkins University, Baltimore, MD, USA

**Keywords:** dry eye syndrome, lacrimal gland, stem/progenitor cells, three-dimensional culture

## Abstract

**Purpose:**

We investigate human lacrimal gland tissue to determine the presence of progenitor cells in this adult human tissue.

**Methods:**

Six human lacrimal gland tissues from donors were collected and stored immediately in the culture medium at 4°C until the next procedure. One part of the lacrimal gland tissue was prepared for immunofluorescence staining and the other part was prepared for primary cell culture. Immunofluorescence analysis was conducted to evaluate cultured lacrimal epithelial phenotype and progenitor cell markers for five passages. Real-time polymerase chain reaction (PCR) was performed to assess proliferation markers in the different passages. Three-dimensional culture and PCR were conducted to determine the differentiation potential of cultured human lacrimal gland cells.

**Results:**

Human lacrimal gland tissue expressed a number of epithelial progenitor cell markers. Precursor cell markers C-Kit, K15, Nestin, and P63 were observed in lacrimal gland tissues. Lacrimal gland epithelial cells were cultured successfully and passaged to P5. The cultured lacrimal gland epithelial cells were positive for pan-cytokeratin (PCK), AQP5, Rab3D, ABCB5, C-kit, K15, Ki67, and P63. Human lacrimal gland cells could form spheroids in vitro and then grow into mini-gland-like structures. PCR results showed proliferation and differentiation capability of those cultured cells.

**Conclusions:**

Human lacrimal gland tissues contain precursor marker-positive cells and marker expression also was detected in ex vivo cultured cells, which showed differentiation capability.

**Translational Relevance:**

Future studies of differentiation in human lacrimal gland tissue may aid in developing stem cell-based therapies for dry eye disease.

## Introduction

The lacrimal gland is a tubuloacinar gland that is critical for ocular health and vision quality. It produces the aqueous components of the tear film, including water, electrolytes, and proteins. The tear film forms a smooth refractive layer over the ocular surface, while lubricating the conjunctiva and cornea, and supporting ocular surface metabolism. The lacrimal gland also serves as a secretory immune system to protect the ocular surface against infection by releasing immunoglobulins into the tears.^1^

In humans, the primary part of lacrimal gland is located within the upper temporal orbit, sending its secretions into an anastomosed duct system that delivers the tear component to the ocular surface. In rodents, however, the lacrimal gland is composed of two lobular structures: one intra- and the other extraorbital.^2^ Study of human lacrimal epithelial cell biology is limited by poor access to human tissue and difficulty with primary cultures; thus, most studies published on lacrimal gland biology are based on rodents; very few have used human samples.^3^,^4^

The lacrimal gland epithelium is composed of three major cell types: acinar, ductal, and myoepithelial cells.^5^ Acinar cells constitute 80% of the lacrimal gland, contributing to the primary secretory apparatus. The secretory ducts lined by cuboidal duct cells connected with the luminal sides of the acinar cells, which constitute 10% to 12% of the lacrimal gland cell population and contribute to approximately 30% of the lacrimal gland fluid secretions. Myoepithelial cells surround the basal part of acinar and ductal cells, which apply pressure to the two kinds of secretory cells to expel the fluid into the duct system. Besides these three epithelial cell types, the lacrimal gland stroma also contains fibroblasts that secrete extracellular matrix, and mast cells that produce histamines and matrix proteins into the interstitial spaces. The regenerative ability of lacrimal gland tissue was reported by several groups.^6^–^8^ However, researchers still debate the roles of specific cellular components in regeneration.

Aqueous-deficient dry eye disease results from impairment of lacrimal gland function, which can progress to corneal ulceration and even vision loss if left untreated. One significant risk factor for dry eye disease is aging, and is associated with lacrimal gland structural and functional changes, characterized by lymphocytic infiltration, atrophied acini, duct obstruction, and decreased protein secretion.^1^ Lacrimal gland dysfunction also can arise from inflammation triggered by the autoimmune attack and undesired environment, as in Sjögren's syndrome and rheumatoid arthritis, and side effects of chemo- and radiation therapies, as well as congenital defects.^9^

Several treatments exist, including punctal occlusion^10^ to reduce tear drainage; anti-inflammatory drugs, such as topical cyclosporine (Restasis; Allergan, Inc., Madison, NJ; Cequa; Sun Pharmaceutical Industries Ltd., Mumbai, India),^11^ and lifitegrast (Xiidra; Shire US Inc, Lexington, MA)^12^; and, most commonly, artificial tears and gels. However, each of these clinical interventions is primarily palliative, and not aimed at curing the underlying lacrimal gland deficiency.^13^

To this end, repair of the damaged lacrimal gland by cell-based therapy or bioengineered replacements can potentially provide long-lasting and physiologic treatments for lacrimal gland dysfunctions.^5^ Ensuring the success of these therapeutic approaches will require a thorough understanding of the cell biology and molecular mechanisms of lacrimal gland development and regeneration.

## Methods

### Human Lacrimal Gland Cell Culture

Human lacrimal gland samples were recovered by SightLife (Seattle, WA) within 24 hours postmortem. The use of human tissue was approved by the institutional review board (IRB) of Johnsons Hopkins University and is in accordance with the tenets of the Declaration of Helsinki. The fresh gland was collected in fetal calf serum (FCS)–rich Dulbecco's modified eagle medium (DMEM) supplemented with antibiotic-antimycotic (Thermo Fisher Scientific, Waltham, MA) in 4°C and transported to the lab, where it was immediately taken for processing.

The lacrimal glands (half portion) were washed and chopped into small segments. The pieces were placed in an Erlenmeyer flask and digested with an enzyme cocktail of Hank's balanced salt solution (Ca^2+^-free, Mg^2+^-free; Millipore Sigma, Burlington, MA) , ethylenediaminetetraacetic acid (EDTA; Millipore Sigma) and a mixture of collagenase (350 U/mL; Gibco 17018-029; Thermo Fisher Scientific), hyaluronidase (300 U/mL; Worthington Biochemical Corp LS02592, Lakewood, NJ), DNase (40,000 U/mL; Millipore Sigma 260913), followed by incubation in a shaking water bath at 37°C for 15 minutes. The supernatant was collected easily after adding 30 mL Ham's nutrient mixture F-12 (Irvine Scientific, Santa Ana, CA). A cell pellet was obtained by centrifugation at 252*g* for 5 minutes. The cells were resuspended by adding 5 mL Ham's solution. To maximize cell recovery, the digestion process was repeated twice. To minimize contamination of nontarget cells, such as, for example, fibroblasts, a 70-μm cell strainer (BD Biosciences, San Jose, CA) and 5% Ficoll (Millipore Sigma) were used. Isolated cells were cultured in DMEM/Nutrient Mixture F-12 (DMEM/F12; Thermo Fisher Scientific) supplemented with 1% insulin-transferrin-selenium (ITS; Thermo Fisher Scientific), 0.1% cholera toxin (Millipore Sigma), 5% KnockOut Serum Replacement (Thermo Fisher Scientific), 10 ng/mL epidermal growth factor (EGF; Thermo Fisher), and 5 μM Y-27632 rho associated coiled-coil protein kinase (ROCK) inhibitor (Cayman Chemical, Ann Arbor, MI).

### Immunofluorescence

For immunofluorescence analysis, harvested lacrimal glands were embedded in optimal cutting temperature (OCT) embedding compound and stored at −80°C. Lacrimal gland sections of 8 μm were cut and placed on slides. Frozen sections were fixed with 100% acetone. Cultured lacrimal gland cells were fixed with 4% paraformaldehyde (Millipore Sigma). After rinsing three times with ×1 phosphate buffered saline containing 0.2% Triton-100 (TPBS; Millipore Sigma), samples were blocked with 5% donkey serum blocker (Millipore Sigma) for 1 hour at room temperature. Samples were immunostained for stem/progenitor cell-related markers, including nestin (Santa Cruz Biotechnology, Dallas, TX), cytokeratin 14 (K14; Santa Cruz Biotechnology), cytokeratin 15 (K15; Santa Cruz Biotechnology), C-Kit (Santa Cruz Biotechnology), ABCG2 (Santa Cruz Biotechnology), ABCB5 (Santa Cruz Biotechnology), and ΔNp63 (Santa Cruz Biotechnology); epithelial cell marker pancytokeratin (PCK; Millipore Sigma); duct cell-specific marker cytokeratin 4 (K4; Millipore Sigma), and myoepithelial marker α-smooth muscle actin (SMA; Millipore Sigma). To determine whether lacrimal gland progenitor cells underwent acinar differentiation, immunostaining for lactoferrin (Abcam, Cambridge, UK) and RAB3B (Santa Cruz Biotechnology) were performed. The cells were incubated with primary antibodies overnight at 4°C in a humidified chamber. After appropriate washing with TPBS three times, the cells were incubated for 1 hour at room temperature with fluorescent dyes Alexa Fluor (AF) 488- or AF568-conjugated secondary antibodies (Thermo Fisher Scientific). Nuclear staining was done with VECTASHIELD Mounting Media (Vector Laboratories, Burlingame, CA). Samples were analyzed under a Zeiss LSM 710 confocal microscope (Carl Zeiss; Oberkochen, Germany).

### Reverse Transcription Quantitative Polymerase Chain Reaction (RT-qPCR)

Total RNA extraction and reverse transcription, the RNeasy mini kit (Qiagen, Germantown, MD) and SuperScript III First-Strand Synthesis System (Thermo Fisher Scientific) were used according to the manufacturers' instructions. Purified total RNA (1 μg) was used for cDNA synthesis, followed by RT-qPCR amplification in a StepOnePlus Real-Time PCR System (Thermo Fisher Scientific) with a Power SYBR Green PCR Master Mix (Thermo Fisher Scientific). Target primers specific for differentiation genes *AQP5*, *K4*, *LAC*, and the primer for reference gene *β-actin* (forward: 5′-GCTATTTGGCGCTGGACTT-3′, reverse: 5′-GCGGCTCGTAGCTCTTCTC-3′) were designed using Primer 3 software.

### Three-Dimensional (3D) Culture

We used a spheroid culture method modified from one for neural stem cells, and then a floating organoid culture method modified from one used for thymic epithelial cells and lacrimal gland epithelium as we reported previously.^6^ Briefly, after isolation, lacrimal gland progenitor cells were resuspended in culture medium as 4 × 10^6^ cells/mL, and 50 μL of cell suspension was seeded in each micro-mold (Microtissues, Inc., Providence, RI). The formed spheroids were cultured in low attachment flasks for one more day, and then seeded into a 15 μL drop of laminin I (6 mg/mL; Trevigen, Inc., Gaithersburg, MD) sitting on a polycarbonate filter floating in serum-free medium for 2 weeks.

### Statistical Analysis

Results were expressed as means ± standard error of the mean (SEM) and were statistically compared by analysis of variance (ANOVA); *P* < 0.05 was considered statistically significant.

## Results

### Human Lacrimal Gland Epithelial Cells Primary Culture

**Figure 1:**
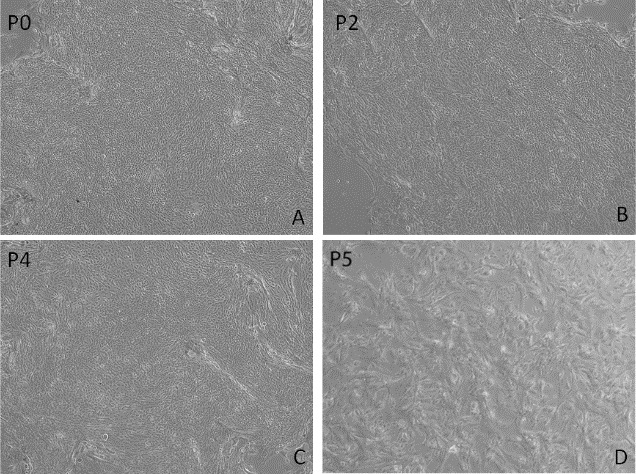
The isolated cells from human lacrimal gland formed a monolayer in P0-P4 (A–C) with epithelial morphology in compact organization, and then be passaged to P5 (D) with a greater diversity of cell shapes.

The enzymatic digestion of the freshly harvested lacrimal gland tissue yielded a cell monolayer in P0 in our culture system with the majority of cells appearing as cube-shaped epithelium (Fig. 1A).The shape of cells did not change much in P2 (Fig. 1B). During the subculture period, cells from four samples could be passaged to P3, and cells from two other sample could be passaged to P4 and P5, respectively. However, although the cells still formed a cuboidal epithelial monolayer in P4 (Fig. 1C), the majority of cell shapes in the culture system were replaced with spindle-shaped fibroblast-like cells in P5 (Fig. 1D).

### Immuno-Phenotype of Native and Cultured Lacrimal Gland Cells

**Figure 2:**
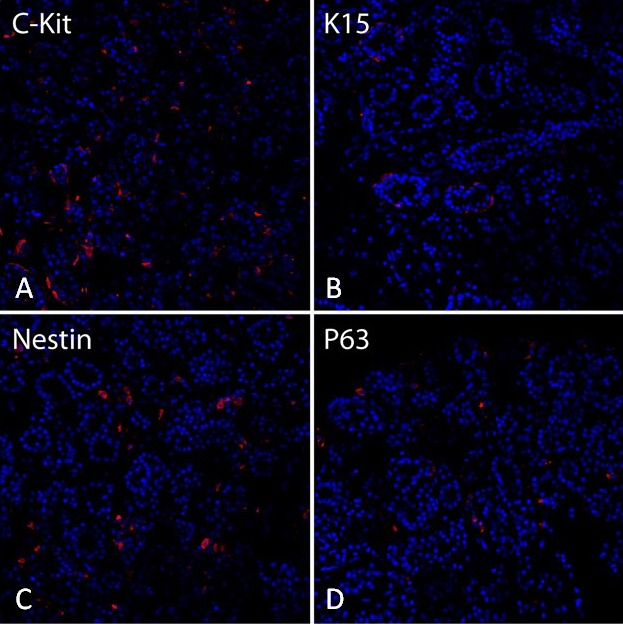
Immunofluorescent staining of human lacrimal gland tissue. C-Kit (A) and nestin (C) staining was visualized scattered around the acini, while K15 (B) and P63 (D) staining was found mainly in the ductal areas.

The epithelial progenitor markers we used in the immunohistochemistry study of the human lacrimal gland samples are illustrated in Figure 2. These OCT-embedded sections showed immunoreactivity for C-Kit, K15, nestin, and P63. Cells positive for C-Kit (Fig. 2A) and nestin (Fig. 2C) are scattered and localized in the tissue's myoepithelial and acinar cells. The staining pattern reveals localization of K15 (Fig. 2B) and P63 (Fig. 2D) mostly in the ductal cells.

**Figure 3:**
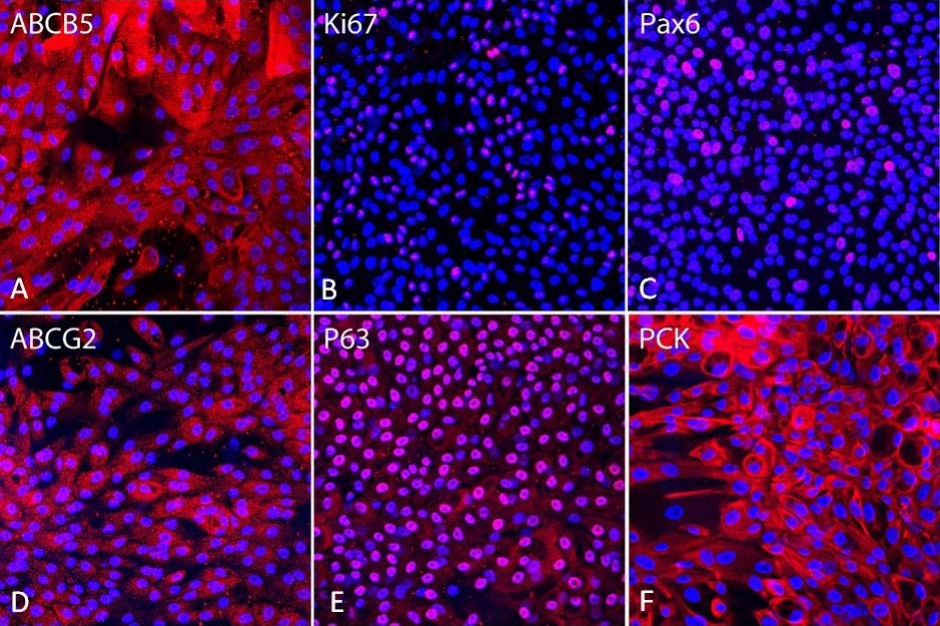
Immunofluorescent staining of the monolayer cells from human lacrimal glands in P1. Most of these cells are ABCB5 (A), Pax6 (C), ABCG2 (D), P63 (E), and PCK (F)–positive. Some of the cells are Ki67 (B)–positive.

The cultures showed that the monolayer cell sheet in P1 (Fig. 3) had a most homogeneous population of cells with immunoreactivity for epithelial markers. The cells with epithelial morphology showed positivity for ABCB5 (Fig. 3A), Pax6 (Fig. 3C), ABCG2 (Fig. 3D), P63 (Fig. 3E), and PCK. Some of those cells also were positive for Ki67 (Fig. 3B). ABCB5, ABCG2, and PCK (Figs. 3A, 3D, 3F, respectively) localized in the cytoplasm, while Ki67, Pax6, and P63 localized in the nucleus (Fig. 3). A minimum of α-SMA positive cells were found in P1 (data not shown).

**Figure 4:**
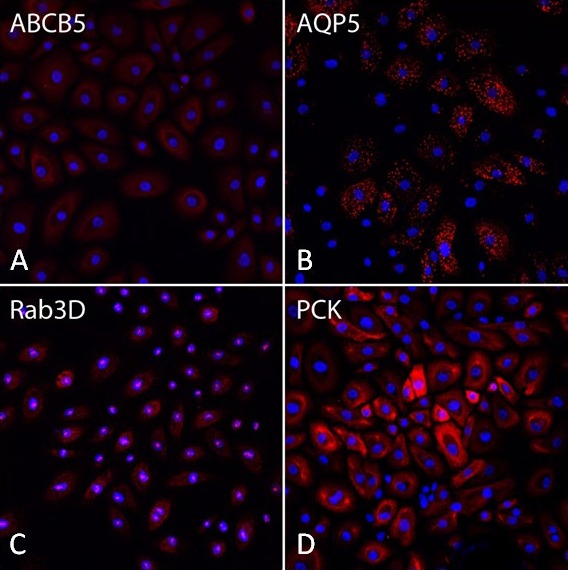
Immunofluorescent staining in monolayer cells from human lacrimal glands in P4. Most of the cells are still ABCB5 (A), AQP5 (B), Rab3D (C), and PCK (D)–positive.

Lacrimal gland cells in P4 showed a different immunoactivity pattern from P1 cells. Although they remained PCK-positive, the Ki67- and P63-positive staining was rarely apparent in those cells (data not shown). The intensity of ABCB5 staining in those cells was much decreased compared to that in P1 cells. Lacrimal gland markers AQP5 and Rab3D were detected in most cells (Fig. 4). Similar to P1, α-SMA–positive cells were hardly found in P4 (data not shown).

### 3D Culture of Human Lacrimal Gland Epithelial Cells

**Figure 5:**
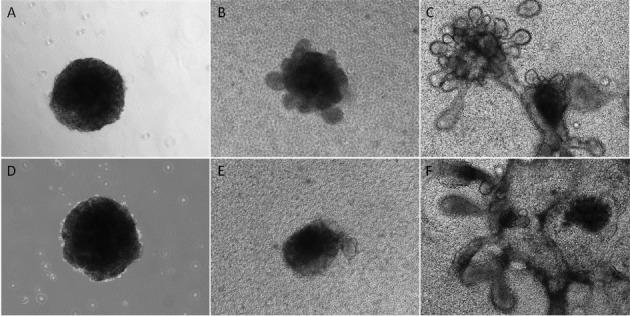
3D culture of human lacrimal gland epithelial cells. Lacrimal gland epithelial cells in P1 formed similarly sized lacrimal spheres in suspension culture (A), and gradually branched out in 2 weeks (days in culture: 3 [B], 14 [C]) to form mini-glands in laminin gel. The spheres from P4 (D) showed branching potential, but the structure is less compact (days in culture: 3 [E], 14 [F]).

**Figure 6:**
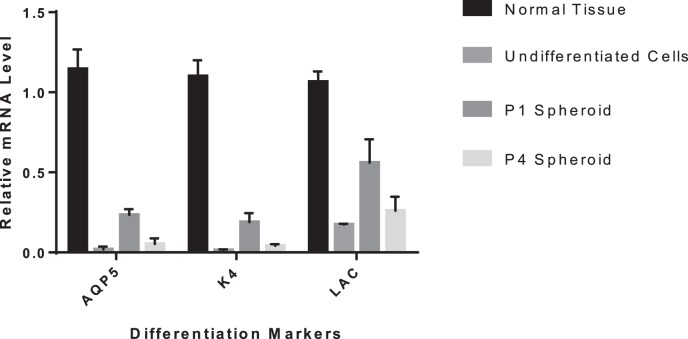
RT-qPCR results of 3D culture of human lacrimal gland spheroids.

Lacrimal gland epithelial cells in P1 and P4 formed similar spheroids by using microtissue micro-molds (Figs. 5A, 5D). For P1 sphere, several small buddings started to sprout out from lacrimal spheres cultured in the laminin gel on day 3 of culture (Fig. 5B), and gradually formed tubular and acinar-like structures by 2 weeks of culture (Fig. 5C, 26.7% in all spheres). For P4 sphere (Fig. 5D), the branching process was less obvious (Fig. 5E) and the cells just formed less compact structures as more spreading layer with several tube-like structures (8.4%, Fig. 5F). RT-qPCR results showed increased gene expression levels of *AQP5*, *K4*, and *LAC* (lacrimal gland differentiation markers) during 3D culture (Fig. 6), especially in the P1 spheroids culture.

## Discussion

The human lacrimal gland is difficult to obtain and few reports exist on its primary culture.^14^ To our knowledge, several groups have attempted to culture human lacrimal gland cells and published their findings in the human lacrimal gland epithelial culture systems.^4^,^15^ A recent study used a sphere-forming assay to develop lacrispheres from human lacrimal gland samples for potential clinical application.^15^ However, no information on the proliferation and differentiation capability of ex vivo monolayer cultured human lacrimal gland epithelial cells has been reported to our knowledge. We characterized the human lacrimal gland by immunostaining of progenitor markers on native tissue and cultured monolayer cells, and further differentiated the lacrimal gland epithelial cells into mini-gland-like structures with potential secretory function.

A sphere-forming assay has been used for salivary gland and lacrimal gland culture. In our previous work, the spheroids from rabbit lacrimal gland could be seeded into decellularized bioscaffold and even developed secretory function ex vivo.^5^ We also demonstrated that monolayer culture using a modified protocol and selection medium could successfully expand the rabbit lacrimal gland progenitor cells from regenerated tissues.^6^ Here, we adopted the culture system from our previous work in monolayer culture with serum-free conditions.

The difficulty in using stem cell biomarkers is that there is no single marker that can be counted upon as a true stem cell marker. To compensate for this shortcoming, a wider range of strategies, such as evaluating the expression of pluripotency and development markers, functional assays, sorting studies, and gene expression signature profiling, usually are used. In our study, the presence of stem-like cells was explored by evaluating the expression of stem cell markers in situ and in cultured cells, proliferation, and evaluation of differentiation potential.

Multipotency is a major characteristic of adult stem cells. Secretory cells, here the acinar cells, compose the majority of cells and are the most important cell types in the functional lacrimal gland. Thus, we tested the differentiation potential of our lacrimal gland epithelial progenitor cells isolated from the human tissues in laminin gel 3-D cultures. As shown in Figures 5 and 6, lacrimal gland progenitor cells in P2 began to form acinotubular structures within 3 days of culture and eventually formed mini-glands in 2 weeks, with the elevated gene expression level of multiple lacrimal gland differentiation markers. However, those cells lost their differentiation potential very quickly, as shown by the incompact structures formed from P4 spheres. This finding might result from limitations in the ex vivo expansion system and limited approaches to verify the differentiation potential (e.g., very limited cell numbers from the beginning of culture and tissue from older donors, inaccessiability in phenotypic analysis in P5 cells, as well as lacking of immunostaining in the 3D culture). The finding might also suffer from the limitation of lacking enough epithelial-mesenchymal interaction during the expansion and differentiation. In future experiments, we may combine the sphere-forming assay with our current culture system to test the differentiation potential and investigate the epithelial-mesenchymal interaction.

## Conclusion

Our study provides very promising, first-of-its-kind evidence for the generation of 3D mini-glands from native human lacrimal gland. The mini-glands contained differentiated, secretory-competent cells. We strongly believe that further validation of the regenerating potential of these lacrimal gland epithelial cells would take us one step closer to potential clinical application of cell therapy-based treatment for severe dry eye.
